# Metrics of Gender Differences in Mortality Risk after Diabetic Foot Disease

**DOI:** 10.3390/jcm12093288

**Published:** 2023-05-05

**Authors:** Giuseppe Seghieri, Elisa Gualdani, Piergiorgio Francia, Ilaria Campesi, Flavia Franconi, Graziano Di Cianni, Paolo Francesconi

**Affiliations:** 1 Epidemiology Unit, Agenzia Regionale Sanità, 50141 Florence, Italy; 2 Faculty of Physiatry, University of Florence, 50121 Florence, Italy; 3 Department of Information Engineering, University of Florence, 50121 Florence, Italy; 4 Laboratorio Nazionale di Farmacologia e Medicina di Genere, Istituto Nazionale Biostrutture Biosistemi, University of Sassari, 07100 Sassari, Italy; 5 Diabetes and Metabolic Diseases Unit, Health Local Unit North-West Tuscany, 57121 Livorno, Italy

**Keywords:** gender differences, diabetic foot disease, mortality risk, first-incident hospitalization, ratio of hazard ratios

## Abstract

Background: The aim of this study was to clarify any gender differences in the mortality risk of people with DFD since patients with diabetic foot disease (DFD) are at a high risk of mortality and, at the same time, are more likely to be men. Methods: From regional administrative sources, the survival probability was retrospectively evaluated by the Kaplan-Meier method and using the Cox proportional-hazards model comparing people with DFD to those without DFD across the years 2011–2018 in Tuscany, Italy. Gender difference in mortality was evaluated by the ratio of hazard ratios (RHR) of men to women after initial DFD hospitalizations (n = 11,529) or in a cohort with prior history of DFD hospitalizations (n = 11,246). Results: In both cohorts, the survival probability after DFD was lower among women. Compared to those without DFD, after initial DFD hospitalizations, the mortality risk was significantly (18%) higher for men compared to women. This excess risk was particularly high after major amputations but also after ulcers, infections, gangrene, or Charcot, with a lower reduction after revascularization procedures among men. In the cohort that included people with a history of prior DFD hospitalizations, except for the risk of minor amputations being higher for men, there was no gender difference in mortality risk. Conclusions: In people with DFD, the overall survival probability was lower among women. Compared to those without DFD after a first DFD hospitalization, men were at higher risk of mortality. This excess risk disappeared in groups with a history of previous DFD hospitalizations containing a greater percentage of women who were older and probably had a longer duration of diabetes and thus becoming, over time, progressively frailer than men.

## 1. Introduction

Diabetic foot disease (DFD) has a significant medical and social relevance for patients carrying a great risk of mortality, comparable to the risk of death associated with cancer [1,2,3,4,5,6,7,8]. Furthermore, DFD is much more prevalent among males [9], who additionally have a greater absolute risk of mortality and cardiovascular complications than women [10]. For these reasons, the increase in the prevalence of DFD among men and its high risk of mortality have led to the impression that men with DFD are at higher risk of death than women [11]. Prior studies on this issue, however, present some evident shortcomings being in the great majority biased by the low number of patients studied and, having mostly considered cohorts coming from diabetic-foot specialized outpatient clinics.

To complicate matters further, there is also to consider that diabetes confers to women a greater relative risk of cardiovascular events such as myocardial infarction or ischemic stroke [12,13]. There are, moreover, several reports suggesting that women with diabetes appear to be disadvantaged when compared with their male counterparts and are less likely to receive treatments for the reduction of the overall risk of cardiovascular diseases as recommended by guidelines [14,15]. Moreover, only a few studies have considered the risk of mortality separately by gender, evaluating patients in two distinct situations: after a first-incident hospitalization for DFD or surviving after prior hospitalizations for DFD. In addition, DFD is a complex clinical picture including a set of complications (ulcers, gangrene, infections, amputations, Charcot disease), each with a different prognostic profile, which again could have different outcomes between genders. This retrospective observational study concerned hospitalizations for DFD, as retrieved by administrative sources in a large population of people with diabetes, followed up for eight years in Tuscany, Italy. It has been designed to evaluate: (a) whether there is a gender difference in the risk of death after DFD, separately examining men and women, (b) gender differences in mortality after different DFD complications, and (c) any gender difference in mortality after a first-incident DFD hospitalization or considering a cohort with prior DFD hospitalizations at baseline.

## 2. Methods

### 2.1. Study Design and Data Source

The population under study consisted of all identified people with diabetes resident in Tuscany, a region of central Italy, as of 1 January 2011, retrospectively followed up until 31 December 2018, as reported in a recent population epidemiological study [16]. In brief, the diagnosis of diabetes was based on the administrative databases at the Regional Health Agency of Tuscany, in Florence, Italy, as previously detailed [17]. This initial population was divided into two cohorts: the first included all individuals who had no previous hospitalizations for DFD complications as of 1 January 2011, or at entry into the study. The second cohort included all individuals with a history of previous DFD hospitalizations at baseline, divided by ICD-9 CM codes: ulcers: 440.23, 707.14-5, Charcot neuro-arthropathy: 713.0, 713.5, 713.8, infections: 6811, 6819, 6826, 6827, 6829, 730.07, 730.17, 730.27, 99.21, gangrene: 440.20, 440.21, 440.22, 440.23, 440.29, 443.9, 785.4, 040.0, 440.24, major and minor lower extremity amputations: 84.10–84.19, and revascularizations (surgical: 39.25, 39.29, endoluminal: 39.50, 39.90). In both cohorts the presence of comorbidities was diagnosed according to Charlson index, scored as 0, 1, or 2.

### 2.2. Statistical Analysis

The incidence of death (all-causes mortality) occurring within the period from 1 January 2011 to 31 December 2018 was retrieved in both cohorts from the database of the regional registry office concerning all deaths in our region. The time-to-event was considered as the interval from the first-ever incident of diabetic foot hospitalizations or from 1 January 2011 to death or until the end of the study.

Survival probability after DFD was evaluated using the Kaplan-Meier survival analysis. In the cohort with first-ever incident DFD hospitalizations, the relative risk of death, comparing those with DFD hospitalization to those without it, was calculated by using the Cox proportional hazards model to assess the hazard ratios (HRs) of all-causes death, separately for men and women. This was also performed considering all DFD complications: 1. major amputations, 2. minor amputations, 3. ulcers, gangrene, infections, or Charcot, and 4. revascularizations. In this analysis, the model was adjusted for age, the Charlson index, and antidiabetic therapy.

In the cohort of those with prior DF hospitalizations at baseline, again after stratifying by gender, HRs of all-causes mortality were calculated using Cox proportional hazards models, comparing those with DFD hospitalizations to those without hospitalizations as the reference group. Additionally, in this case the time-to-event was considered the interval from 1 January 2011 to death, or to the end of the study, after adjusting for age, Charlson index, and antidiabetic therapy.

To measure the difference in relative risk of death between genders, we measured the ratio of hazard ratios (HR), men to women (RHR), calculating the 95% CIs as suggested by Woodward [18].

All data were anonymized and based on administrative datasets, preventing any disclosure of patients’ identities as well as any other sensitive information. Because of such formal protection, no informed consent or any approval by an Ethics Committee was required, according to current national and regional rules.

All analyses were performed using SAS ver. 9.3, SAS Institute Inc., Cary, NC, USA.

## 3. Results

The main characteristics of the two cohorts under study are shown in Table 1. Both cohorts contained about the same number of DFD hospitalizations (11,246 vs. 11,529), and men were more represented, even if less so among those without previous hospitalizations at baseline (61.6% vs. 66.1%; *p* < 0.05). The mean age was higher in the cohort with first-incident DF hospitalizations (74 ± 10 years vs. 71 ± 11 years; *p* < 0.05) and was higher among men than women in the cohort with a first-incident DFD, being conversely higher among women in the cohort with prior hospitalizations at baseline (Table 1). Most DFD hospitalizations were due to ulcers, gangrene, infections, or Charcot in both cohorts, followed by revascularization procedures, with lower rates for amputations, both major and minor, especially in the cohort with prior hospitalizations at baseline. The prevalence of comorbidities, as suggested by the Charlson index, as well as the prevalence of therapy with insulin, were higher in the cohort with prior hospitalizations (Table 1). Finally, the mortality rate was significantly higher among women in the cohort with prior hospitalizations: 0.58 per 1000 p-y (95% CI 0.55–0.60) vs. 0.48 (95% CI 0.47–0.49) and conversely was higher among men, compared to women in the cohort of first-incident DFD hospitalizations (0.78 per 1000 p-y (95% CI 0.74–0.81) vs. 0.52 (95% CI 0.50–0.54); *p* = 0.0001 for both.

**Figure 1:**
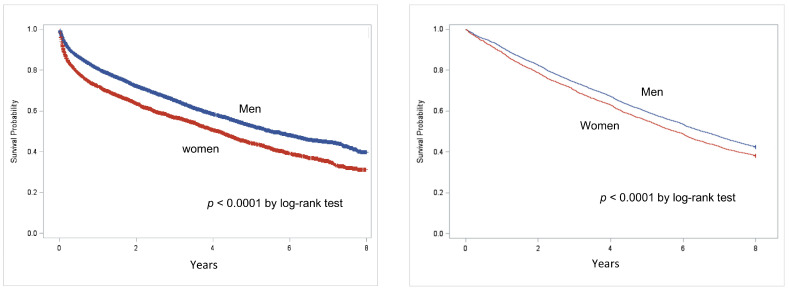
Kaplan-Meier survival curves in populations after first-incident DFD hospitalization (**left**) or in the cohort, including people with prior hospitalizations for DFD at baseline (**right**) by gender (red: women, blue: men).

The survival probability in people with DFD, as evaluated using the Kaplan-Meier analysis, is reported in Figure 1. Survival probability was reduced among women as compared to men (*p* < 0.0001 by log-rank test) in both cohorts, with a mortality rate approaching 50% at 5 years for both genders. The impact on early mortality was higher after a first-incident DFD hospitalization among women than among men n = 429; 5.79 (5.27–6.37) vs. n = 656; 5.51 (5.10–5.94) per 1000 p-y; *p* < 0.05.

**Figure 2:**
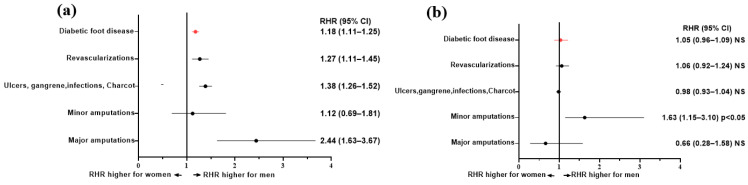
Ratio of adjusted HRs: RHR, men to women of all-causes mortality by DFD complications comparing people with or without DFD after a first-ever DFD hospitalization (**a**) or in cohort with prior DFD hospitalizations at baseline (**b**). In red: RHR for all complications.

The adjusted HRs of death comparing people with DFD hospitalizations vs. those without DFD hospitalizations, as obtained by Cox regression models, are reported in Table 2. In the cohort of those with a first-incident DFD hospitalization, men were at significantly higher adjusted risk of mortality when compared with women: HR: 4.34 (95% CI 4.15–4.53) vs. 3.68 (95% CI 3.54–3.82); *p* < 0.05. This difference, however, vanished in the cohort with prior baseline DFD hospitalizations: HR: 1.60 (95% CI 1.53–1.66) for men and 1.56 (95% CI 1.51–1.62) for women, *p* = NS. The ratio between HRs and RHR was accordingly significantly higher by 18% for men vs. women, by (*p* = 0.001) after a first-ever hospitalization and resulted as non-significant in the cohort with prior hospitalizations (Table 2 and Figure 2). After a first-incident DFD hospitalization, major amputations, ulcers, gangrene, infections, or Charcot had a higher risk of mortality in both genders that was significantly higher among men compared to women, as testified by the higher RHR, especially for major amputations: 2.44 (95% CI 1.63–3.67); *p* < 0.05. Interestingly, revascularizations had a significant protective effect against mortality of approximately 40% among females: 0.59 (0.55–0.64) *p* = 0.001, lessening to approximately 25% HR: 0.75 (0.67–0.83) *p* = 0.001 among men, resulting in a reduced beneficial effect on mortality risk of about 27% among men: 1.27 (95% CI 1, 11–1.45); *p* < 0.05 (Table 2 and Figure 2). However, all these differences were no more present in the cohort with prior baseline hospitalizations, except for minor amputations, which maintained a higher RHR (1.63 95% CI 1.15–3.10) for men vs. women (Table 2 and Figure 2).

## 4. Discussion

This study is the first, to our knowledge, to detail a gender difference in the risk of death after hospitalizations for DFD through a suitable statistical methodology in a large population. What is confirmed by the present study is that DFD was much more prevalent among men [9,19,20,21], who, as known, are at higher absolute risk of cardiovascular events [12,13]. This greater incidence among men is moreover reaffirmed, for DFD, by a recent and comprehensive review on the etiology and epidemiology of diabetic foot ulcers [9] as well as by other studies regarding amputations in DFD [22,23,24]. The issue could be, consequently, closed with the conclusive suggestion that the risk of death in people with DFD is overall greater among men [11]. No prior population study or review, however, tried to investigate gender differences, if any, distinguishing between mortality risk after prevailing or first-incident DFD hospitalizations, as well as after any single DFD complication such as amputations, ulcers, infections, ischemic lesions, or revascularizations. In addition, most prior studies were not population-based, being often made of small groups of selected patients, and adjustment for comorbidities was not always possible. Furthermore, not many previous studies have focused on revascularizations, which this study confirms as predictive of longer survival in both sexes [25]. Further, a key point to be taken into account is that even if men have a greater absolute risk of being affected with cardiovascular events, diabetes confers a greater relative risk for cardiovascular diseases (myocardial infarction, stroke, or peripheral artery disease) to females compared to men [12,13,26,27]. This study, based on a population followed up for eight years, suggests that from a gender perspective, the risk of mortality in people with DFD has several facets. First, the survival rate was significantly reduced for women in people with DFD, with a mortality rate approaching 40–50% after 5 years for both genders in both cohorts and with a greater mortality rate in the first years after a first-incident DFD hospitalization. Further, comparing people with and without DFD, we used a methodology apt to catch risk differences between genders [18]. According to this method, we studied populations separately by gender and used the ratio between relative risks, here Hazard Ratios, as a tool to quantify differences [18]. This study, accordingly, confirmed that men, compared to people without DFD, were at higher risk of mortality than women by about 18% after a first DFD hospitalization and that such excess risk of mortality was more than twice as high for men, especially after major amputations, remaining significantly increased after hospitalizations due to ulcers, infections, or Charcot HR:1.38 (95% CI 1.26–1.52); *p* = 0.001. This gender difference, however, was no more evident in the cohort with a history of prior DFD hospitalizations, suggesting, in this case, a progressive increase in the relative risk of death among women, as also reflected by the loss of the protective effect exerted by revascularizations [25] among women. The protective effect of revascularizations after a first-incident hospitalization could also be related to the reduction in rates of both major and minor amputations in the cohort with a prior history of DFD hospitalizations. Previously, our study, moreover, demonstrated that in people with a prior history of DFD hospitalizations, females with amputations or with peripheral arterial disease were at greater risk of subsequent adverse cardiovascular events than those with ulcers, infections, or Charcot [28], further underlining the greater relative risk of cardiovascular events conferred by diabetes among women. Similarly, in a previous paper, we showed that even after a first-incident DFD hospitalization, foot ulcers, infections, or Charcot predicted a greater risk of cardiovascular events among women compared to men [29]. The reason why, in the presence of the previous history of DFD hospitalizations, the excess risk of mortality among men seems to be decreased is most likely due to the fact that this cohort included a greater number of women, who were moreover more elderly, essentially including all survivors after prior DFD hospitalizations. Further, considering age as a proxy for the duration of diabetes, women with prior DFD hospitalizations at baseline presumably had more longstanding diabetes. In addition, the relative risk for diabetes complications seems to be greater for women than for men also because women are currently less likely than men to receive treatment for diabetes as well as for the reduction of cardiovascular risk, as recommended by guidelines [14,15], with excess risk of cardiovascular events becoming more evident over time [30]. Interestingly, moreover, it has been described that women seem to delay amputation compared to men [31], possibly delaying post-amputation complications, including increased death risk. Additionally, women are likely to have a higher probability of ulcer healing as a first event, so eventually postponing all more severe consequences of foot ulcers, especially in the case of recurrences [32]. In conclusion, the mortality risk associated with DFD, as explored by an appropriate methodology, largely shows significant differences between genders. The crude unadjusted mortality rate after both prevailing and first-incident DFD hospitalization was higher among women. Men, moreover, when compared to those without DFD, appeared to be most disadvantaged after a first hospitalization for DFD. In the cohort with a baseline history of prior hospitalizations, instead, the women seem to become increasingly frail over time with a progressive rise in mortality risk, thus nullifying the advantage over men. Summarizing, according to this retrospective observational study, with the presence of DFD, the eight-year survival in our population was markedly reduced, especially for women. After comparing people hospitalized for DFD with those without DFD hospitalizations, the absolute risk of being affected with DFD was much higher among men. The relative mortality risk, however, seems to be modulated and varied depending on whether to consider the cohort after a first-incident DFD hospitalization or the cohort with a history of prior DFD hospitalizations. After a first DFD hospitalization for men, compared to women, they were at a significantly higher risk of mortality of about 20%. This significant excess risk for men occurred especially after major amputations but, albeit to a lesser extent, also after ulcers, infections, gangrene, or Charcot. The reduction in risk of mortality given by revascularization procedures was significantly lower for men. This excess risk disappeared in people with a history of previous DFD hospitalizations, except for minor amputations. This may be because the cohort of people with a history of prior DFD hospitalizations included a higher percentage rate of women who were older than men and, therefore, more likely to have a longer duration of diabetes, thus appearing progressively frailer than men.

### 4.1. Limitations and Strengths of the Study

Due to the administrative source of data utilized in this study, the main limitation comes from the impossibility of having clinical details at hand and, consequently, adjusting results for any possible unmeasured risk factors. A further limitation is the exclusion of non-hospitalized patients usually managed in outpatient settings. In this case, however, we presume to have not missed all the most serious cases requiring hospitalization and, therefore, with a greater mortality risk. The strengths of our study may be found in the large sample of the population, without the limitation of being chosen from selected patients coming from specialized outpatient clinics as well as in the solid methods used to measure gender differences.

### 4.2. Conclusions

In conclusion, the key message of this study is that even if men are at higher risk of developing DFD with its most ominous outcomes, women should require greater caution, either after a first DFD hospitalization or with a history of DFD in the past. Further, these differences between men and women recommend, in our opinion, the development of guidelines for the management of DFD also from a gender perspective.

## Figures and Tables

**Table 1:** Characteristics of the two cohorts of patients with previous DFD hospitalizations or after first-ever DFD hospitalizations, by gender.

	with Previous DFD Hospitalizations		*p*	after First-Ever DFD Hospitalizations		*p*
	Men	Women		Men	Women	
Age (yr) (mean ± SD)	73 ± 10	77 ± 11	0.0001	75 ± 10	69 ± 11	0.0001
Charlson Index (mean ± SD)	1.7 ± 0.6	1.6 ± 0.6	NS	1.03 ± 0.90	1.0 ± 0.98	NS
DFD hospitalizations No (%)	7437 (66)	3809 (34)		7108 (62)	4421 (38)	
Major Amputations No (%)	29 (0.3)	10 (0.4)		73 (1.0)	70 (1.6)	
Minor Amputations No (%)	52 (0.7)	34 (0.9)		199 (3.0)	107 (2.4)	
Ulcers, gangrene, infections, Charcot No (%)	6253 (84)	3307 (87)		4961 (70)	3265 (74)	
Revascularizations No (%)	1103 (15)	458 (12)		1875 (26)	979 (22)	
Insulin No (%)	1567 (21)	939 (25)	0.0001	795 (11)	588 (13)	NS
Oral No (%)	2847 (38)	1268 (33)	0.0001	3103 (43)	1842 (42)	NS
Insulin/oral No (%)	1156 (15)	667 (17)	NS	895 (12)	591 (13)	NS
None No. (%)	1867 (25)	935 (24)	NS	2385 (33)	1400 (32)	NS
No. of deaths (%)	4279 (57)	2354 (62)		2850 (56.7)	2178 (49.2)	
Mortality rate per 1000 p-y (95% CI)	0.48 (0.47–0.49)	0.58 (0.55–0.60)	0.0001	0.78 (0.74–0.81)	0.52 (0.50–0.54)	0.0001

**Table 2:** Adjusted HR and Ratio of HRs (RHR), men to women of all-causes mortality after all DFD hospitalizations (**a**), after first-incident DFD hospitalization by complications (**b**), or in the cohort including people with prior hospitalizations for DFD at baseline by complications (**c**), comparing people with or without DFD.

(a) HR by gender and RHR men to women (95% CI) of mortality after first-incident DFD or in cohort with prior hospitalizations at baseline						
	Men	*p*	Women	*p*	RHR	*p*
	HR (95% CI)		HR (95% CI)			
After first-incident DFD hospitalizations (n = 11,529)	4.34 (4.15–4.53)	0.001	3.68 (3.54–3.82)	0.001	1.18 (1.11–1.25)	<0.05
After prior DFD hospitalizations (n = 11,246)	1.60 (1.53–1.66)	0.001	1.56 (1.51–1.62)	0.001	1.05 (0.96–1.09)	NS
**(b) HR by gender and RHR men to women (95% CI) of mortality after first-incident hospitalization for DFD complications by gender**						
	**Men**	***p***	**Women**	***p***	**RHR**	***p***
	**HR (95% CI)**		**HR (95% CI)**			
Major Amputations (n = 143)	3.97 (2.98–5.12)	0.001	1.62 (1.18–2.16)	0.001	2.44 (1.63–3.67)	<0.05
Minor Amputations (n = 306)	1.17 (0.85–1.55)	NS	1.04 (0.83–1.29)	NS	1.12 (0.69–1.81)	NS
Ulcers, gangrene, infections, Charcot (n = 8226)	1.26 (1.12–1.32)	0.001	0.91 (0.87–0.95)	0.001	1.38 (1.26–1.52)	<0.05
Revascularizations (n = 2854)	0.75 (0.67–0.83)	0.001	0.59 (0.55–0.64)	0.001	1.27 (1.11–1.45)	<0.05
**(c) HR by gender and RHR men to women (95% CI) of mortality in cohort with prior hospitalizations for DFD complications**						
	**Men**	***p***	**Women**	***p***	**RHR**	***p***
	**HR (95% CI)**		**HR (95% CI)**			
Major Amputations (n = 39)	1.91 (1.05–3.15)	0.001	2.87 (1.36–5.11)	0.001	0.66 (0.28–1.58)	NS
Minor Amputations (n = 86)	1.75 (1.11–2.58)	0.001	1.07 (0.63–1.66)	NS	1.63 (1.15–3.10)	<0.05
Ulcers, gangrene, infections, Charcot (n = 9560)	1.63 (1.57–1.69)	0.001	1.66 (1.59–1.74)	0.001	0.98 (0.93–1.04)	NS
Revascularizations (n = 1561)	1.25 (1.15–1.35)	0.001	1.17 (1.02–1.33)	0.001	1.06 (0.92–1.24)	NS

## Data Availability

Data are available upon reasonable request with permission of Agenzia Regionale Sanità Toscana, Florence, Italy.
